# Effect of ketamine on cellular immunity and inflammation in patients who undergo laparoscopic colon cancer surgery: a retrospective study

**DOI:** 10.3389/fphar.2025.1562122

**Published:** 2025-08-21

**Authors:** Jiajia Ji, Haiping Zhang, Lixia Zhou, Chunlan Fan, Ligen Dai, Shaolei Mao

**Affiliations:** Anesthesiology Department, Bayingolin Mongolian Autonomous Prefecture People’s Hospital, Korla, China

**Keywords:** ketamine, laparoscopic colon cancer surgery, cellular immunity, inflammatory cytokines, adverse reactions

## Abstract

**Objective:**

Anesthesia during the surgery impairs immune systems. Ketamine is an anesthetic with immune protective effects. This study intended to investigate the effect of a ketamine-involved anesthetic regimen on cellular immunity and inflammatory cytokines in patients who undergo laparoscopic colon cancer surgery.

**Methods:**

This retrospective study screened 60 patients who underwent laparoscopic colon cancer surgery with an anesthesia regimen involving ketamine (N = 30, ketamine group) or not (N = 30, opioid group). Data on surgery-related parameters, blood pressure, heart rate (HR), blood routine examination parameters, cluster of differentiation (CD) 3^+^ and CD4^+^ T cells, inflammatory cytokines, and adverse reactions were retrieved. Time points were defined as entering the operating room (T1), 5 min after anesthetic induction (T2), the end of surgery (T3), and 24 h after surgery (T4).

**Results:**

The 1 h-postoperative pain score (*P* < 0.001) and length of stay (*P* < 0.001) were lower in ketamine group than in opioid group. Blood pressure and HR from T1 to T4 were more stable in ketamine group than in opioid group. Neutrophils (*P* < 0.001), CD3^+^ (*P* < 0.001) and CD4^+^ T cells (*P* = 0.002) at T4 were higher in ketamine group than in opioid group. Interleukin (IL)-10 at T3 (*P* < 0.001) and T4 (*P* < 0.001) were higher, while IL-6 at T3 (*P* < 0.001) and T4 (*P* < 0.001) were lower in ketamine group than in opioid group. There was no discrepancy in incidences of adverse reactions between groups.

**Conclusion:**

A ketamine-involved anesthetic regimen seems to be correlated with the improvement of cellular immunity and inflammation with a tolerable safety profile. However, more studies with a prospective, randomized, controlled design are needed to verify this finding and draw a solid conclusion.

## Introduction

Colon cancer is one of the most frequent and deadly malignancies around the world, and it has been a serious problem threatening public health (Alrushaid et al., 2023; Katsaounou et al., 2022). Currently, surgical resection is the main treatment option for colon cancer patients (Shinji et al., 2022). However, anesthesia during the surgery impairs the immune system, expressed as cellular immune suppression and excessive proinflammatory immune response, which leads to an increased risk of disease recurrence and postoperative infections (Ackerman et al., 2021; Brogi and Forfori, 2022; Wang et al., 2023). The addition of anesthetic drugs with the capability of attenuating the impairment of the immune system may contribute to improving the prognosis of patients who undergo colon cancer surgery.

Ketamine, an antagonist of the glutamate N-methyl-D-aspartate (NMDA) receptor, is a common drug in anesthesia (Coles et al., 2023). Current evidence has suggested that ketamine has immune protective effects, which are specifically manifested as protecting cellular immunity and inhibiting inflammation (Hou et al., 2016; Johnston et al., 2023; Liao et al., 2014; Zhang et al., 2021). For example, a study suggested that ketamine increased the ratio of the cluster of differentiation (CD) 4^+^/CD8^+^ T cells and the percentage of regulatory T (Treg) cells *in vitro* (Hou et al., 2016). Some studies also showed that ketamine inhibited inflammation through the nuclear factor erythroid 2-related factor 2, adenosine monophosphate-activated protein kinase/mammalian target of rapamycin, or nuclear factor kappa B pathway (Cao et al., 2022; Xiao et al., 2022; Zhao et al., 2023). From the clinical perspective, it was disclosed that ketamine reduced interleukin (IL)-6 and tumor necrosis factor-alpha in patients who received abdominal surgery (Beilin et al., 2007). Based on the above studies, it is hypothesized that a ketamine-involved anesthetic regimen may contribute to protecting the immune system in patients who undergo colon cancer surgery. However, there is a lack of relevant studies.

Therefore, this study intended to explore the effect of a ketamine-involved anesthetic regimen on cellular immunity and inflammatory cytokines in patients who underwent laparoscopic colon cancer surgery.

## Methods

### Patients

In this retrospective study, a total of 60 patients who underwent laparoscopic colon cancer surgery with general anesthesia were screened between January 2022 and January 2023. The inclusion criteria included: 1. Patients who underwent radical resection surgery; 2. Patients aged 45–80 years; 3. Patients who had no prior radiotherapy, chemotherapy, or other anti-tumor treatments; 4. Patients classified as American Society of Anesthesiologists (ASA) physical status I-II. The exclusion criteria included: 1. Patients with uncontrolled or untreated hypertension (systolic/diastolic blood pressure (SBP/DBP) exceeding 180/110 mmHg), or those with significantly elevated intracranial pressure; 2. Patients with untreated or inadequately treated hyperthyroidism; 3. Patients with immune dysfunction or those who received immunotherapy prior to surgery. The approval was gained from the Ethics Committee of Bayingolin Mongol Autonomous Prefecture People’s Hospital (No. BZRMYY (2024)-6). All patients or their families signed the informed consent. The flowchart was shown in Supplementary Figure S1.

### Anesthetic drug regimen

Patients would be assigned to the ketamine or opioid groups according to the treatment regimen that they received.

#### Ketamine group

Anesthesia induction: Ketamine 1.0–3.0 mg/kg, sufentanil 0.3–0.6 μg/kg, midazolam 0.05 mg/kg, propofol 1.5–2.0 mg/kg, and cisatracurium 0.15–0.25 mg/kg. After loss of consciousness, assisted ventilation was provided, followed by tracheal intubation after 3 min.

Anesthesia maintenance: Ketamine 0.5 mg/kg/h and propofol 3–5 mg/kg/h were infused continuously. Cisatracurium 6–8 mg/h was administered intermittently to maintain muscle relaxation.

#### Opioid group

Anesthesia induction: Sufentanil 0.3–0.6 μg/kg, midazolam 0.05 mg/kg, propofol 1.5–2.0 mg/kg, and cisatracurium 0.15–0.25 mg/kg. After loss of consciousness, assisted ventilation was provided, followed by tracheal intubation after 3 min.

Anesthesia maintenance: Remifentanil 10–20 μg/kg/h and propofol 3–5 mg/kg/h were infused continuously. Cisatracurium 6–8 mg/h was administered intermittently to maintain muscle relaxation.

### Assisted ventilation information

All patients received assisted ventilation using a volume-controlled mode. Tidal volume was set at 6–8 mL/kg of predicted body weight, with a respiratory rate of 12–14 breaths per minute. Ventilation parameters were adjusted based on end-tidal carbon dioxide (ETCO_2_) levels to maintain, ETCO_2_ within the target range of 30–35 mmHg.

### Data collection and conversion

Age, sex, underlying diseases, ASA classification, morphine equivalent dose, and surgery-related parameters were collected. The morphine equivalent dose was converted according to the following formula: 1 mg sufentanil≈700 mg morphine; 1 mg remifentanil≈150 mg morphine. Blood pressure and heart rate (HR) at the following time points were retrieved: entering the operating room (T1), 5 min after anesthetic induction (T2), the end of surgery (T3), and 24 h after surgery (T4). Blood routine examination parameters, CD3^+^, CD4^+^, CD8^+^ T cells, NK cells, and B cells percentages data were collected, which were detected at T1 and T4. The inflammatory cytokines were also collected, which were measured at T1, T3, and T4. Adverse reactions that patients experienced were also retrieved.

### Flow cytometry for lymphocyte subset analysis

Peripheral venous blood samples (2 mL) were collected into EDTA-K_2_ anticoagulant tubes and processed within 4 h of collection. Lymphocyte subsets, including CD3^+^, CD4^+^, and CD8^+^ T cells, NK cells, and B cells, were analyzed using multiparametric flow cytometry. Briefly, 100 µL of whole blood was incubated with fluorochrome-conjugated monoclonal antibodies against CD3, CD4, CD8, CD16, CD56, and CD19 (BD Biosciences, San Jose, CA, United States) for 20 min at room temperature in the dark. Red blood cells were lysed using BD FACS™ Lysing Solution according to the manufacturer’s instructions, and the remaining leukocytes were washed twice with phosphate-buffered saline (PBS). The samples were resuspended in 500 µL PBS and acquired on a BD FACSCanto™ II flow cytometer (BD Biosciences, United States). Data were analyzed using FlowJo software, version X.0.7 (BD Biosciences, United States). Lymphocyte subsets were expressed as percentages of total lymphocytes and as absolute counts, calculated from corresponding complete blood count results.

### Measurement of serum TNF-α, IL-6, and IL-10

Serum concentrations of TNF-α, IL-6, and IL-10 levels were measured using enzyme-linked immunosorbent assay (ELISA) kits from Wuhan Bionly Biotechnology Co., Ltd. (Wuhan, China), performed in duplicate according to the manufacturer’s protocols.

### Statistics analyses

Data processing was performed using SPSS v.26.0 (IBM, United States). Between-group comparisons were made using the Student’s t-test (normal distribution variables), Wilcoxon rank sum test (non-normal distribution variables), *χ* ^*2*^ test (counting variable), and Fisher’s exact test (counting variable, expected frequency <5 in any cell of a 2 × 2 contingency table). Within-group comparisons were made using paired sample t-test (normal distribution variables, two time points), Wilcoxon signed-rank test (non-normal distribution variables, two time points), repeated measures ANOVA (normal distribution variables, three or more time points), and Friedman test (non-normal distribution variables, three or more time points). A *P* < 0.05 indicated statistical significance.

## Results

### Comparisons of clinical characteristics

There were 13 (43.3%) females and 17 (56.7%) males in the ketamine group, with a mean age of 58.4 ± 11.8 years. The opioid group had 14 (46.7%) females and 16 (53.3%) males, with a mean age of 60.4 ± 12.8 years. No difference was observed in age, sex, underlying diseases, or ASA classification between groups (all *P* > 0.05). However, the morphine equivalent dose was lower in the ketamine group than in the opioid group [median interquartile range (IQR): 21.0 (17.5–21.0) versus 230.0 (214.1–257.4) mg; *P* < 0.001] (Table 1).

**TABLE 1:** Clinical characteristics of patients undergoing laparoscopic colon cancer surgery.

Characteristics	Ketamine group (N = 30)	Opioid group (N = 30)	*P* Value
Age (years), mean ± SD	58.4 ± 11.8	60.4 ± 12.8	0.519
Sex, n (%)			0.795
Female	13 (43.3)	14 (46.7)	
Male	17 (56.7)	16 (53.3)	
Underlying diseases, n (%)			1.000
No	21 (70.0)	26 (70.0)	
Yes	9 (30.0)	9 (30.0)	
ASA classification, n (%)			1.000
I	2 (6.7)	3 (10.0)	
II	28 (93.3)	27 (90.0)	
Morphine equivalent dose (mg), median (IQR)	21.0 (17.5–21.0)	230.0 (214.1–257.4)	<0.001

SD, standard deviation; ASA, american society of anesthesiologists; IQR, interquartile range.

### Comparisons of surgery-related parameters

No discrepancy was observed in operating time (168.0 ± 18.0 versus 171.9 ± 17.8 min) or postoperative extubation time [median (IQR): 17.0 (13.0–22.0) versus 16.5 (11.8–19.8) min] between groups (both *P* > 0.05). The 1 h-postoperative pain score based on the visual analog scale was lower in the ketamine group than in the opioid group [median (IQR): 1.0 (0.0–1.0) versus 2.0 (1.0–3.0); *P* < 0.001]. Meanwhile, the length of stay was also lower in the ketamine group than in the opioid group [median (IQR): 9.5 (8.8–11.0) versus 12.0 (10.8–14.3) days; *P* < 0.001] (Table 2).

**TABLE 2:** Surgery-related parameters.

Items	Ketamine group (N = 30)	Opioid group (N = 30)	*P* Value
Operating time (min), mean ± SD	168.0 ± 18.0	171.9 ± 17.8	0.398
Postoperative extubation time (min), median (IQR)	17.0 (13.0–22.0)	16.5 (11.8–19.8)	0.378
1 h-postoperative pain score (VAS), median (IQR)	1.0 (0.0–1.0)	2.0 (1.0–3.0)	<0.001
Length of stay (days), median (IQR)	9.5 (8.8–11.0)	12.0 (10.8–14.3)	<0.001

SD, standard deviation; IQR, interquartile range; VAS, visual analog scale.

The information about disease history and their previous drug regimens were shown in the Supplementary Table S1. Beside, the subgroup analysis was carried out based on the underlying disease. It indicated that operating time and postoperative extubation time were similar between two groups in both patients with and without underlying diseases subgroups. The 1 h-postoperative pain score was lower in ketamine group compared with opioid group in both patients with and without underlying diseases subgroups. However, the length of stay was shorter in the ketamine group compared with opioid group in subgroup of without underlying diseases, but remained similar between two groups in subgroup of with underlying diseases (Supplementary Table S2). These findings indicated that these patients seemed to benefit more from ketamine in the subgroup of patients without underlying diseases.

### Comparisons of blood pressure and heart rate

Within-group comparisons showed that SBP was decreased from T1 to T2, then gradually increased from T2 to T4 in the ketamine group and the opioid group (both *P* < 0.001). DBP did not vary at each assessment point in the ketamine group (*P* = 0.061), but it declined from T1 to T2, increased from T2 to T3, and then remained stable from T3 to T4 in the opioid group (*P* < 0.001). HR did not change from T1 to T4 in the ketamine group (*P* = 0.063). However, in the opioid group, HR was declined from T1 to T2, and increased continuously from T2 to T4 (*P* < 0.001).

**FIGURE 1:**
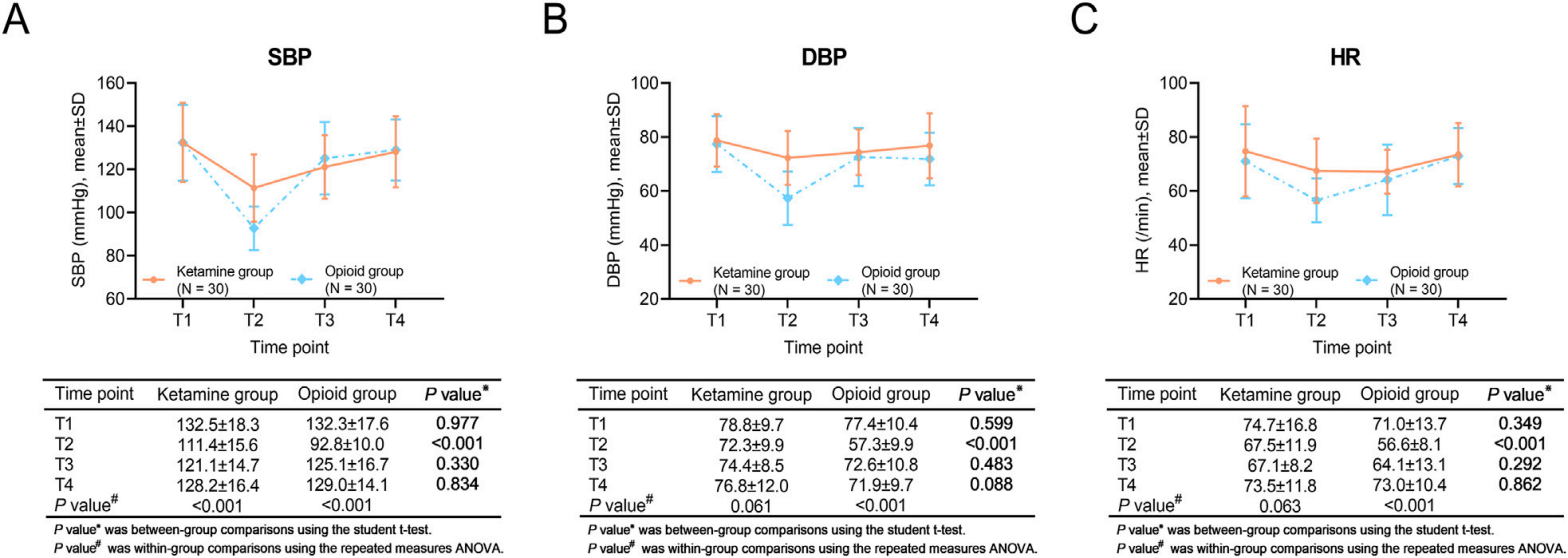
SBP, DBP, and HR at different time points in ketamine and opioid groups. Within-group and between-group comparisons of SBP **(A)**, DBP **(B)**, and HR **(C)**.

Regarding between-group comparisons, SBP, DBP, and HR at T2 were higher in the ketamine group than in the opioid group (all *P* < 0.001). No discrepancy was revealed in SBP, DBP, or HR at other time points between the two groups (all *P* > 0.05) (Figures 1A–C).

### Comparisons of blood routine examination parameters

Within-group comparisons revealed that white blood cells (WBC), neutrophils (NE), neutrophil-to-lymphocyte ratio (NLR), and platelet-to-lymphocyte ratio (PLR) were elevated, while lymphocytes (LY) were reduced at T4 compared to T1 in the ketamine group and the opioid group (all *P* < 0.001). Meanwhile, platelet counts (PLT) were reduced at T4 versus T1 in the ketamine group (*P* = 0.017), but PLT did not vary between T1 and T4 in the opioid group (*P* = 0.178).

Between-group comparisons showed NE at T4 was higher in the ketamine group than in the opioid group (*P* < 0.001). However, there was no variation in WBC, PLT, LY, NLR, or PLR at T1 or T4 between the ketamine group and the opioid group (all *P* > 0.05) (Table 3).

**TABLE 3:** Blood routine examination parameters of patients undergoing laparoscopic colon cancer surgery at T1 and T4.

Parameters	Ketamine group (N = 30)	Opioid group (N = 30)	*P* value^⋇^
WBC (◊10^9^/L), median (IQR)			
T1	6.4 (5.6–6.7)	6.2 (5.4–6.7)	0.578
T4	10.5 (9.6–12.9)	9.9 (9.2–11.1)	0.129
*P* value^#^	<0.001	<0.001	
PLT (◊10^9^/L), median (IQR)			
T1	227.0 (204.0–250.0)	223.0 (210.5–288.3)	0.970
T4	184.0 (164.3–221.3)	199.0 (174.0–252.0)	0.128
*P* value^#^	0.017	0.178	
NE (%), median (IQR)			
T1	65.1 (55.9–66.9)	57.9 (55.8–66.0)	0.230
T4	86.1 (83.6–90.2)	81.0 (79.2–81.6)	<0.001
*P* value^#^	<0.001	<0.001	
LY (%), median (IQR)			
T1	29.8 (26.1–33.3)	33.3 (24.1–34.1)	0.269
T4	7.5 (5.4–11.0)	8.1 (5.8–10.8)	0.241
*P* value^#^	<0.001	<0.001	
NLR, median (IQR)			
T1	2.2 (1.7–2.6)	1.9 (1.7–2.2)	0.259
T4	11.5 (7.3–16.4)	10.0 (7.7–13.9)	0.086
*P* value^#^	<0.001	<0.001	
PLR, median (IQR)			
T1	117.6 (95.4–133.1)	133.6 (98.5–157.4)	0.280
T4	223.0 (150.6–283.5)	255.8 (186.7–334.4)	0.164
*P* value^#^	<0.001	<0.001	

P value^⋇^ was between-group comparisons using the Wilcoxon rank sum test.

P value^#^ was within-group comparisons using the Wilcoxon signed-rank test.

T1, entering the operating room; T4, 24 h after surgery; WBC, white blood cells; IQR, interquartile range; PLT, platelet counts; NE, neutrophils; LY, lymphocytes; NLR, neutrophil-to-lymphocyte ratio; PLR, platelet-to-lymphocyte ratio.

### Comparisons of CD3^+^ and CD4^+^ T cells

Regarding within-group comparisons, CD3^+^ T cells were descended at T4 compared to T1 in the ketamine group and the opioid group (both *P* < 0.001). CD4^+^ T cells were also reduced at T4 compared to T1 in the ketamine group (*P* = 0.010) and the opioid group (*P* < 0.001).

**FIGURE 2:**
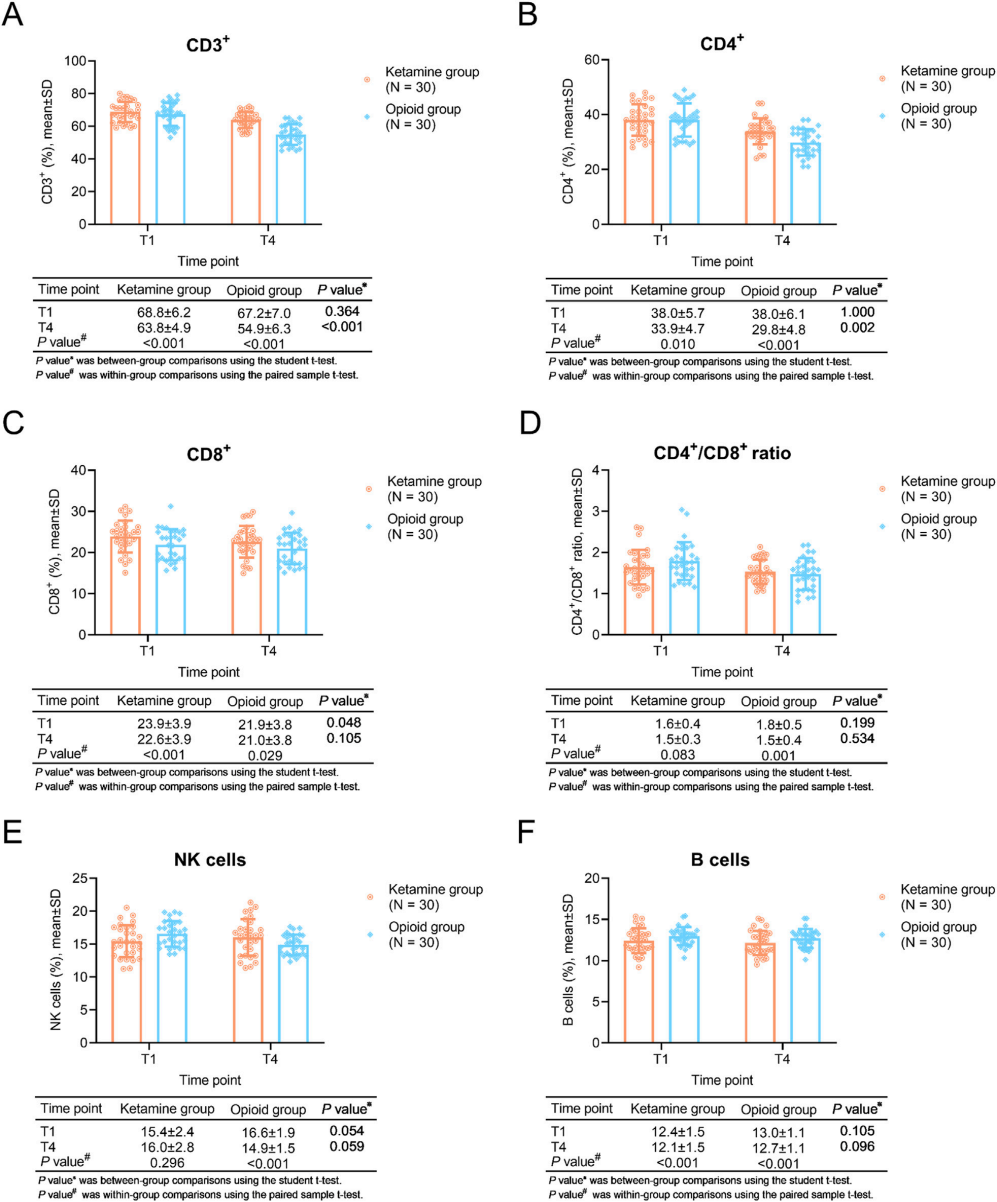
T cells, NK cells, and B cells at different time points in ketamine and opioid groups. Within-group and between-group comparisons of CD3^+^ T cells **(A)**, CD4^+^ T cells **(B)**, CD8^+^ T cells **(C)**, CD4^+^/CD8^+^ T cells **(D)**, NK cells **(E)**, and B cells **(F)**.

Between-group comparisons showed that CD3^+^ T cells (*P* < 0.001) and CD4^+^ T cells (*P* = 0.002) at T4 were higher in the ketamine group than in the opioid group (Figures 2A,B).

The CD8^+^ cells was higher in ketamine group than opioid group at T1 (*P* = 0.048), while it remained similar between groups at T4 (*P* = 0.105). In terms of the CD4^+^/CD8^+^ ratio, it was similar between two groups both at T1 and T4 (both *P* > 0.05) (Figures 2C,D).

The NK cells remained unchanged in ketamine group (*P* = 0.296), while it was decreased in opioid group (*P* < 0.001), even though there was no difference between groups both at T1 and T4 (both *P* > 0.05) (Figure 2E). Furthermore, the B cells were reduced after the treatment in ketamine group and opioid group (both *P* < 0.001), while it remianed unchanged between groups both at T1 and T4 (both *P* > 0.05) (Figure 2F).

### Comparisons of inflammatory cytokines

Within-group comparisons showed that IL-6 was gradually increased from T1 to T4 in both ketamine and opioid groups (both *P* < 0.001). IL-10 was elevated from T1 to T3, then kept stable from T3 to T4 in the ketamine group (*P* < 0.001); however, it was increased from T1 to T3 and decreased from T3 to T4 in the opioid group (*P* = 0.002).

**FIGURE 3:**
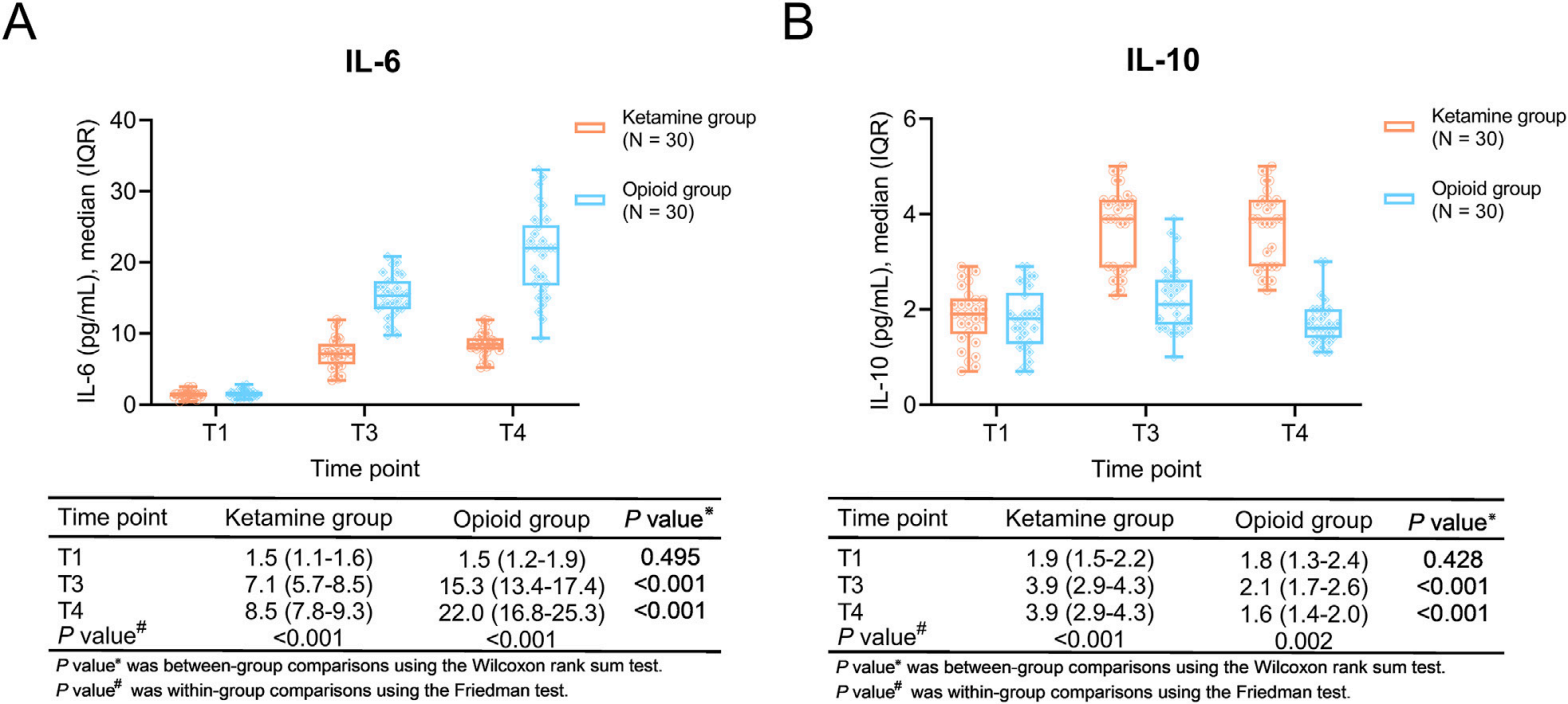
Inflammatory cytokines at different time points in ketamine and opioid groups. Within-group and between-group comparisons of IL-6 **(A)** and IL-10 **(B)**.

In between-group comparisons, IL-6 at T3 and T4 were lower in the ketamine group than in the opioid group (both *P* < 0.001). IL-10 at T3 and T4 were higher in the ketamine group than in the opioid group (both *P* < 0.001) (Figures 3A,B).

CRP and TNF-a were increased both in ketamine group and opioid group (all *P* < 0.01), while there were no difference between groups both at T1 and T4 (all *P* > 0.05, Supplementary Table S3). In terms of PCT, it was decrased in ketamine group, while increased in opioid group (both *P* < 0.001); the PCT was similar between groups at T1 (*P* = 0.209), while it was lower in ketamine group compared with opioid group (*P* < 0.001, Supplementary Table S3).

## Comparisons of adverse reactions

The incidences of hypersomnia, delirium, and nausea were 10.0%, 6.7%, and 3.3% in the ketamine group, while they were 6.7%, 3.3%, and 6.7% in the opioid group. There was no discrepancy in the incidences of these adverse reactions between groups (all *P* > 0.05) (Table 4).

**TABLE 4:** Adverse reactions.

Adverse reactions	Ketamine group (N = 30)	Opioid group (N = 30)	*P* Value
Hypersomnia, n (%)	3 (10.0)	2 (6.7)	1.000
Delirium, n (%)	2 (6.7)	1 (3.3)	1.000
Nausea, n (%)	1 (3.3)	2 (6.7)	1.000

## Discussion

Our study showed that the baseline characteristics of the two groups were described as similar. The rationale of similar baseline characteristics between groups was as follows: these two regimens were the common regimens in our hospital. There was not too much restriction when making the decision to choose the regimen, which caused similar baseline characteristics between groups. On the other hand, despite balanced baseline characteristics, only age, sex, underlying diseases, and ASA classification were collected. It was unclear whether other information might differ between groups, potentially introducing hidden confounders. Therefore, a further prospective design was needed.

Moreover, during the surgery period, blood pressure and HR were more stable in the ketamine group than in the opioid group. Even though ketamine showed certain cardiovascular protective effects. This might be affected by the usage of intra-operative catecholamines or other vasoactive agents. In the current study, the most frequently used vasoactive agent is norepinephrine, followed by ephedrine. However, due to the retrospective study design, the data on the usage and dosage were not recorded in the current study. Therefore, this cardiovascular protective effect of ketamine needs to be verified by further prospective studies.

Besides, 1 h-postoperative pain and length of stay were lower in the ketamine group than in the opioid group. This might be due to the fact that ketamine inhibited central sensitization by blocking the NMDA receptor, which prevented opioid-related activation of pronociceptive systems, thus attenuating opioid-caused hyperalgesia (Ma et al., 2023). Overall, the above results of our study showed that ketamine provided a certain benefit in reducing pain and length of stay. Besides, in this study, the observed reduction in morphine equivalent dose in the ketamine group was likely attributable to the analgesic properties of ketamine, which acts as an NMDA receptor antagonist and has well-established opioid-sparing effects. Ketamine’s ability to reduce central sensitization and enhance pain control may reduce the need for additional opioids, thereby accounting for the lower cumulative opioid dose in this group. However, this difference in opioid consumption between the two groups may also represent a potential source of bias in interpreting the downstream immune-related outcomes. Specifically, since opioids, particularly in high doses, are known to exert immunosuppressive effects, the higher opioid exposure in the opioid group may have independently influenced T-cell responses, including the observed reductions in CD4^+^ T cells and the normalization of CD8^+^ levels over time. As such, it becomes challenging to fully isolate the immunomodulatory effects of ketamine from the confounding influence of differing opioid exposure. Moreover, since analgesic protocols were not strictly matched in terms of total analgesic load or standardized titration schedules, variability in pain control or analgesic responsiveness between groups may further complicate the interpretation of immune outcomes. Future studies may consider matching cumulative analgesic doses more closely or including a control arm with equivalent analgesic efficacy but differing mechanisms to better delineate the direct effects of ketamine on immune function.

The impairment of the immune systems of patients during the surgery period, expressed as cellular immune suppression and excessive proinflammatory immune responses, is an important issue (Xu et al., 2022; Zhou et al., 2018). CD3^+^ and CD4^+^ T cells are the main T lymphocytes that mediate cellular immune function status (Chopp et al., 2023; Sun et al., 2023). IL-6 and IL-10 are considered primary inflammatory cytokines in the response to surgery (Li et al., 2023). Herein, our study found that CD3^+^and CD4^+^ T cells were reduced and IL-6 was increased at 24 h after surgery versus entering the operating room in both ketamine and opioid groups. These results might be explained by the following: Surgery-caused injury and anesthesia could impair the immune system (Bohne et al., 2023; Wall et al., 2019). Notably, CD3^+^ T cells, CD4^+^ T cells, and IL-10 were higher, but IL-6 was lower at the end of surgery or 24 h after surgery in the ketamine group than in the opioid group. These findings revealed that ketamine exerted cellular immune protection and anti-inflammatory effects in patients who underwent laparoscopic colon cancer surgery. This might be because: Ketamine inhibited central sensitization processes by blocking the NMDA receptor, which produced preemptive analgesia effects and reduced postoperative stress response, thereby protecting the immune function of patients (Beilin et al., 2007; Miner, 2008). Moreover, ketamine decreased the need for opioid requirement, thus attenuating the immunosuppressive effect. Regarding blood routine examination parameters, our study found that WBC, NE, NLR, and PLR, PLT were increased, but LY was decreased at 24 h after surgery versus entering the operating room in both ketamine and opioid groups. However, NE was higher in the ketamine group than in the opioid group. These results further indicated that ketamine attenuated the impairment of the immune system. Furthermore, At baseline (T1, prior to surgery), CD8^+^ T cell counts were significantly higher in the ketamine group compared to the opioid group, indicating a potential group imbalance before treatment and representing a methodological limitation. This elevated baseline level suggests that the ketamine group may have had a relatively heightened cytotoxic T cell response even before intervention. However, by 24 h postoperatively (T4), no significant difference in CD8^+^ T cell levels was observed between the two groups, implying that ketamine may have a stronger suppressive effect on CD8^+^ T cells compared to opioids, helping to normalize the elevated levels seen at baseline. Interestingly, despite the dynamic changes in CD4^+^ and CD8^+^ subsets, the CD4^+^/CD8^+^ ratio remained stable between groups at both time points, suggesting that the overall balance of helper and cytotoxic T cells was preserved. These findings suggest that ketamine may have a regulatory effect on cytotoxic T cell responses, potentially offering immune-modulating benefits in the perioperative setting.

Previous studies also report the effect of ketamine on immune outcomes, including the NK cells, IL-6, TNF-α, CRP, etc (Cho et al., 2021; Ostović et al., 2025; Toleska et al., 2023; Wang et al., 2023). For instance, one study reports that the change of NK cells, IL-6, TNF-α, and CRP is not different between the ketamine and control (normal saline) group. However, these findings were inconsistent with the current study. The explaination might be as follows: in Jin Sun Cho et al. study, the dose of ketamine was low (0.25 mg/kg ketamine 5 min before the start of surgery, followed by an infusion 0.05 mg/kg/hr) which was lower than that in our study (ketamine 1.0–3.0 mg/kg for induction followed by an infusion ketamine 0.5 mg/kg/h) (Cho et al., 2021). Therefore, this inconsistent result might be derived from the different doses of ketamine. In another study carried out by Helena Ostović et al., it shows that WBC count, IL-6, and IL-8 remain unchanged after the treatment with ketamine. This difference might also be derived from the different dose of ketamine. In detail, Helena Ostović et al.'s study describes a ketamine regimen of 0.5 mg/kg followed by a continuous infusion of 0.2 mg/kg/h, which is also lower than the current study. Hence, in our opinion, the different findings might be derived from the different doses of ketamine (Ostović et al., 2025). However, this hypothesis needs further exploration.

Although ketamine has shown certain clinical benefits in protecting the immune system, adverse events caused by ketamine still cannot be ignored (Feeney and Papakostas, 2023). In our study, the incidence of adverse reactions did not vary between the ketamine group and the opioid group. Moreover, in our study, the adverse reactions in the ketamine group were hypersomnia, delirium, and nausea. Previous studies exhibited that the most frequent adverse reactions of ketamine included nausea, vomiting, and dizziness in patients who received surgery (Murphy et al., 2021; Srinivasarangan et al., 2024). The results of our study were partly similar to these studies (Murphy et al., 2021; Srinivasarangan et al., 2024), indicating a favorable safety profile of ketamine.

The limitations of our study were as follows: (1) The sample size was relatively small, with a total of 60 cases. The small sample raised concerns about statistical power, increased the risk of type I and type II errors, and further limited the generalizability of the findings. Future studies with a larger sample size were required for verification. (2) Our study did not assess the effect of different doses of ketamine in patients who underwent laparoscopic colon cancer surgery, and future studies were required to determine the optimal dose of ketamine administration in these patients. (3) Our study only explored the effect of ketamine from the beginning of surgery to 24 h after surgery. However, anesthetic techniques might affect the long-term outcomes of cancer patients who received surgery (Murphy et al., 2023). Thus, future studies should consider investigating the long-term influence of ketamine in patients who underwent laparoscopic colon cancer surgery. (4) As a retrospective study, its inherent limitations should be noted, such as the choice of anesthetic drug and the selection bias that existed. A further randomized controlled trial was needed to verify the study’s findings.

## Conclusion

In conclusion, a ketamine-involved anesthetic regimen seems to be correlated with the improvement of cellular immunity and inflammation with a tolerable safety profile. However, more studies with a prospective, randomized, controlled design are needed to verify this finding and draw a solid conclusion.

## Data Availability

The original contributions presented in the study are included in the article/Supplementary Material, further inquiries can be directed to the corresponding author.

## Supplementary material

SUPPLEMENTARY FIGURE S1 The flowchart of the current study.
