# Supplementary material for: Effect of ketamine on cellular immunity and inflammation in patients who undergo laparoscopic colon cancer surgery: a retrospective study
Source: Front Pharmacol. 2025 Aug 21;16:1562122. doi: 10.3389/fphar.2025.1562122 (PMC12408674; doi:10.3389/fphar.2025.1562122)
Supplement: Supplementary file 1 [file Supplementaryfile1.docx]

**Supplementary Table 1.** Information on disease histories.

| Characteristics | Ketamine group  (N = 30) | Opioid group  (N = 30) | Previous drug regimens |
| --- | --- | --- | --- |
| Hypertension, n (%) | 4 (13.3) | 4 (13.3) | Nifedipine controlled-release tablets, 30 mg, qd;  Amlodipine besylate, 5 mg, qd |
| Coronary heart disease, n (%) | 2 (6.7) | 2 (6.7) | Atorvastatin, 10-80 mg, qd;  Wen xin ke li, 5 g, tid;  Dan can di wan, 10 pills, tid;  Metoprolol tartrate tablets, 25-50 mg, bid or tid;  Aspirin enteric-coated tablets, 100-300 mg, qd |
| Diabetes mellitus, n (%) | 1 (3.3) | 1 (3.3) | Metformin hydrochloride and glibenclamide tablets, 2 pills, bid;  Insulin glargine injection, 20 IU, qd |
| Hypothyroidism, n (%) | 1 (3.3) | 0 (0.0) | Levothyroxine sodium tablets, 50 μg, qd |
| Unknown, n (%) | 1 (3.3) | 2 (6.7) | Unknown |
