# Supplementary material for: Effect of ketamine on cellular immunity and inflammation in patients who undergo laparoscopic colon cancer surgery: a retrospective study
Source: Front Pharmacol. 2025 Aug 21;16:1562122. doi: 10.3389/fphar.2025.1562122 (PMC12408674; doi:10.3389/fphar.2025.1562122)
Supplement: Supplementary file 2 [file Supplementaryfile3.docx]

**Supplementary Table 3.** Other inflammatory markers of patients undergoing laparoscopic colon cancer surgery at T1 and T4.

| Parameters | Ketamine group  (N = 30) | Opioid group  (N = 30) | *P* value^⋇^ |
| --- | --- | --- | --- |
| CRP (mg/L), median (IQR) |  |  |  |
| T1 | 4.16 (3.09-5.27) | 3.25 (2.65-5.22) | 0.539 |
| T4 | 4.43 (3.07-5.84) | 3.61 (2.95-5.65) | 0.478 |
| *P* value^#^ | <0.001 | 0.008 |  |
| TNF-α (pg/L), median (IQR) |  |  |  |
| T1 | 0.97 (0.89-1.08) | 0.99 (0.89-1.14) | 0.706 |
| T4 | 1.01 (0.96-1.13) | 1.04 (0.98-1.33) | 0.306 |
| *P* value^#^ | <0.001 | <0.001 |  |
| PCT (%), median (IQR) |  |  |  |
| T1 | 0.09 (0.08-0.11) | 0.08 (0.07-0.11) | 0.209 |
| T4 | 0.08 (0.07-0.09) | 0.11 (0.09-0.13) | <0.001 |
| *P* value^#^ | <0.001 | <0.001 |  |

*P* value^⋇^ was between-group comparisons using the Wilcoxon rank sum test.

*P* value^#^ was within-group comparisons using the Wilcoxon signed-rank test.

T1, entering the operating room; T4, 24 hours after surgery; CRP, C-reactive protein; IQR, interquartile range; TNF-α, tumor necrosis factor-alpha; PCT, procalcitonin.
