# Supplementary figures and images for: Effect of ketamine on cellular immunity and inflammation in patients who undergo laparoscopic colon cancer surgery: a retrospective study
Source: Front Pharmacol. 2025 Aug 21;16:1562122. doi: 10.3389/fphar.2025.1562122 (PMC12408674; doi:10.3389/fphar.2025.1562122)

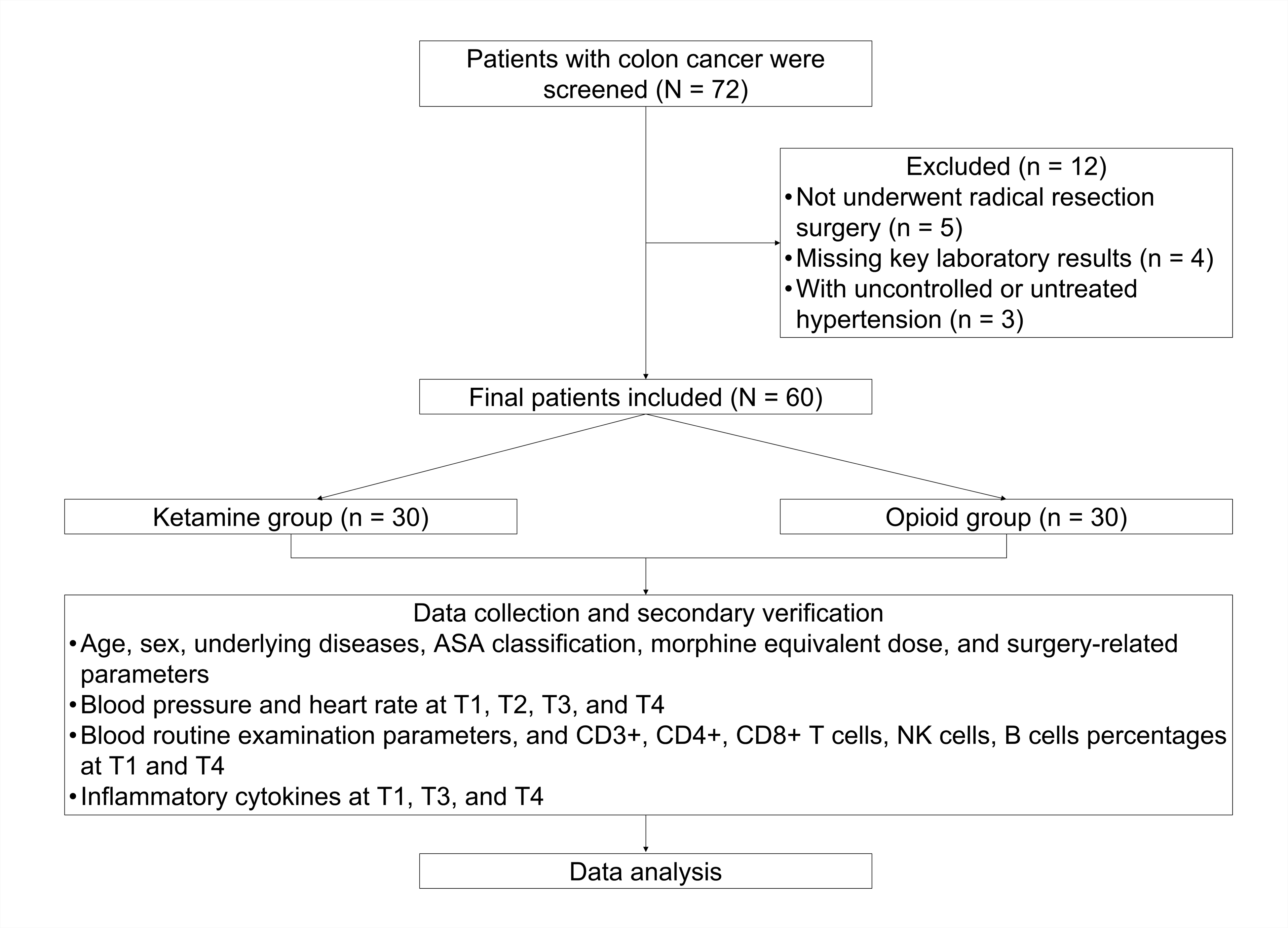

Supplement: Supplementary file 3 [file Image1.tif]
