# Supplementary material for: Effect of ketamine on cellular immunity and inflammation in patients who undergo laparoscopic colon cancer surgery: a retrospective study
Source: Front Pharmacol. 2025 Aug 21;16:1562122. doi: 10.3389/fphar.2025.1562122 (PMC12408674; doi:10.3389/fphar.2025.1562122)
Supplement: Supplementary file 4 [file Supplementaryfile2.docx]

**Supplementary Table 2.** Subgroup analysis of surgery-related parameters.

| Items | Without underlying diseases | | | With underlying diseases | | |
| --- | --- | --- | --- | --- | --- | --- |
|  | Ketamine group  (n = 21) | Opioid group  (n = 21) | *P* value | Ketamine group  (n = 9) | Opioid group  (n = 9) | *P* value |
| Operating time (min), mean±SD | 168.2±18.1 | 173.1±17.3 | 0.374 | 167.4±19.0 | 169.1±19.6 | 0.857 |
| Postoperative extubation time (min), median (IQR) | 17.0 (12.0-24.0) | 17.0 (12.0-20.5) | 0.715 | 18.5 (15.0-21.3) | 16.0 (9.0-21.5) | 0.334 |
| 1 h-postoperative pain score (VAS), median (IQR) | 1.0 (0.0-1.0) | 2.0 (1.0-2.0) | <0.001 | 1.0 (0.3-1.8) | 2.0 (1.5-4.5) | 0.007 |
| Length of stay (days), median (IQR) | 9.0 (8.0-11.0) | 13.0 (11.0-15.0) | <0.001 | 10.0 (9.0-11.5) | 11.0 (10.0-13.5) | 0.105 |

SD, standard deviation; IQR, interquartile range; VAS, visual analog scale.
